# Phylogenic evolution of beat perception and synchronization: a comparative neuroscience perspective

**DOI:** 10.3389/fnsys.2023.1169918

**Published:** 2023-05-31

**Authors:** Jin-Kun Huang, Bin Yin

**Affiliations:** ^1^Laboratory for Learning and Behavioral Sciences, School of Psychology, Fujian Normal University, Fuzhou, Fujian, China; ^2^Department of Applied Psychology, School of Psychology, Fujian Normal University, Fuzhou, Fujian, China

**Keywords:** beat perception and synchronization, musicality, comparative cognition, phylogenic evolution, vocal learning hypothesis, neural synchrony oscillations

## Abstract

The study of music has long been of interest to researchers from various disciplines. Scholars have put forth numerous hypotheses regarding the evolution of music. With the rise of cross-species research on music cognition, researchers hope to gain a deeper understanding of the phylogenic evolution, behavioral manifestation, and physiological limitations of the biological ability behind music, known as musicality. This paper presents the progress of beat perception and synchronization (BPS) research in cross-species settings and offers varying views on the relevant hypothesis of BPS. The BPS ability observed in rats and other mammals as well as recent neurobiological findings presents a significant challenge to the vocal learning and rhythm synchronization hypothesis if taken literally. An integrative neural-circuit model of BPS is proposed to accommodate the findings. In future research, it is recommended that greater consideration be given to the social attributes of musicality and to the behavioral and physiological changes that occur across different species in response to music characteristics.

## Introduction

Music, as defined by Aristotle, was the singing behavior of birds, specifically bird calls. By the time of Darwin, people began to draw analogies between “music” in birds and humans, believing it to be the result of evolution (Darwin, 1871). People once believed that only birds produced musical behavior, “But for humans, birds are perhaps Nature’s only musicians’” (Scholes, 1938, p. 107). However, later research found that marine mammals also produce similar musical behavior, refuting this view (Ralls et al., 1985; Janik and Slater, 1997; Payne, 2000). Many researchers have proposed different hypotheses about the puzzle of music in evolution. For example, Darwin speculated that human musical behavior originated from a biological adaptive trait and that music evolved to attract the opposite sex, with language originating from previously developed musical ability. Other researchers have proposed the null hypothesis that music is just a spandrel for human evolution, a useless byproduct of other evolved abilities with no adaptive function and not involving direct selection for musical ability (Pinker, 1997). However, other researchers believe that music is an important developmental condition in biological evolution, with a specific adaptive purpose, and propose other adaptive hypotheses, including sexual selection (Miller, 2000), advertising male coalitions (Hagen and Bryant, 2003), its role in mother-infant relationships (Fitch, 2006; Mehr and Krasnow, 2017), and its role in enhancing social cohesion in human populations (Wallin et al., 2001; Merker et al., 2009). Unlike scholars who believe that music has adaptive traits, some thinkers believe that human music is a cultural invention built on brain circuits that evolved for other reasons (Pinker, 1997). The debate over the origin of music has continued to drive music-related research. Here, we need to clarify a concept: music cognition is not a single psychological phenomenon, but involves a series of different, interacting psychological processes (Fitch, 2015). Based on this concept, researchers distinguish music as a highly culturally dependent structure (Nettl, 2015), and musicality as a set of psychological abilities underlying basic musical behaviors. In the following discussion, we will mainly describe musicality.

As Tinbergen (1963) described, the problem of the adaptive significance of behavior is only one type of problem regarding the evolution of that behavior. Another important issue involves phylogeny: what is the cross-species and cross-time behavioral history? With the rise of cross-species research in music cognition, many exciting turning points have occurred in the evolution of music research (Fitch, 2015; Honing et al., 2015). This cross-species research can help us gain deeper insight into the phylogeny (evolutionary history), behavior, and physiological constraints behind the biological abilities (called musicality) underlying music cognition. We know that music cognition involves many different abilities, including octave equivalence, consonance preference, saliency of timbre, and rhythm synchronization, all of which may not have appeared simultaneously during evolution. Just as the concept of pluralism implicit in Honing et al. (2015) view of the origin of musicality suggests, the different components of music cognition are likely to have evolved independently, resulting in the possibility of multiple musical origins in humans and animals.

## Beat perception and synchronization

Beat perception and synchronization (BPS) is a form of entrainment that refers to the tight temporal synchronization between periodic movement and auditory rhythms in the time domain (Patel, 2021). This ability is a prominent aspect of human musical behavior and forms the basis of dance (Patel, 2010). Infants exhibit rhythmic characteristics early in life, suggesting a relationship with the evolution of musicality (Winkler et al., 2009; Fujii et al., 2014; Cirelli and Trehub, 2019). Darwin once proposed the idea that “the perception of musical rhythm and beat, if not the enjoyment of it, is common to all animals and undoubtedly depends on their common physiological characteristics of the nervous system” (Darwin, 1871). This is an intuitively appealing view, as rhythm is typically considered the most fundamental aspect of music and is increasingly recognized as a basic organizational principle of brain function (Buzsáki, 2006). Consistent with Darwin’s idea, Patel (2014) suggests that the neural prerequisites for beat matching may be ancient and widely present in evolution. One of these neural prerequisites is neural oscillation, which is widespread in the brains of animals (Large, 2008; Large and Snyder, 2009; Fitch, 2012). When groups of neurons in the brain fire synchronously, neural oscillation occurs, which is an inherent principle of brain function. Large and Snyder (2009) also proposed a theory of music beat perception based on a universal neural mechanism, called the neural resonance theory, which posits that pulse and rhythm correspond to neural rhythms that synchronize with the sound rhythm, affecting timing expectations, attention, and motor coordination. According to this theory, beat perception occurs when non-linear oscillations in the neural system entrain to external rhythmic stimuli.

The viewpoint that BPS exists in most species is in stark contrast to the belief held by some researchers that BPS ability is limited to a small subset of species. One of the current mainstream theories is the “vocal learning and rhythmic synchronization hypothesis,” which suggests that BPS relies on a specialized auditory-motor forebrain circuit that initially evolved to serve complex vocal learning in animals (Patel, 2006). In complex vocal learning, animals require auditory input to develop their normal species-specific vocalizations, and this input can form an auditory template to guide the development of the animal’s own vocalizations (Tyack, 2020). However, such complex vocal learning is not found in all species, and currently known vocal learners are mainly cetaceans, pinnipeds, primates–including humans, and some birds, mainly songbirds, parrots, and hummingbirds.

The vocal music learning hypothesis differs from the neural resonance theory in its ability to make clear and falsifiable predictions. This hypothesis posits that neural changes in auditory-motor circuitry, driven by the evolution of vocal learning, laid the foundation for the capacity to synchronize movement to the beat of music (Patel, 2006). Vocal learning is limited to a small number of animal groups. In the early days of empirical research on this hypothesis, many results were found to be consistent with the hypothesis. For example, Zarco et al. (2009) found that macaques did not synchronize their tapping with the metronome during training compared to human subjects. Although the macaques were able to perform the task of tapping in time with the stimulus signal after a long period of training, they often did not synchronize their tapping but responded with a delay of several 100 ms. Moreover, compared to macaques, human subjects performed the task more easily and could accurately predict the beat time and synchronize their tapping. Findings on parrots (vocal learners) also support the vocal learning hypothesis. In a study of rhythm synchronization in tiger kea parrots, Ai Hasegawa et al. (2011) found that the animal subjects could match their tapping behavior to stimuli of different rhythms, and similar results were also found in Patel et al. (2009a,b) study of sulfur-crested cockatoos (Cacatua galerita eleonora).

Different research findings have been reported in studies of primates. Studies on chimpanzees and bonobos have shown that they exhibit moderate vocal flexibility but are unable to perform complex vocal learning (Fitch, 2010). In contrast, research by Hattori et al. (2013) has demonstrated that chimpanzees (Pan troglodytes) can synchronize their tapping actions with a metronome under certain conditions. However, this rhythmic synchronization is not possible for synchronization with other ranges of rhythms, which is a crucial feature of beat perception and synchronization. Research on marine animals also challenges the hypothesis. Cook et al. (2013) found that after training, sea lions (Zalophus californianus) can resonate and synchronize their movements with various types of stimuli, including music and rhythms. Sea lions are not vocal learners, but they are related to seals and walruses, which are vocal learners. Therefore, researchers suggest that further investigation is needed to determine whether sea lions possess some degree of vocal learning ability, and whether their trained rhythmic synchronization ability is a result of their vocal learning capacity. This issue has yet to be studied further.

The above studies, although they have raised some degree of skepticism about the vocal learning hypothesis, still require more experimental data support. In studies on primates, although they can synchronize rhythms within a specific range, there is no empirical research on their speed flexibility. In the study of sea lions, due to their phylogenetic relationship, it is still uncertain whether their rhythm synchronization ability may be caused by their unproven vocal learning ability. Yet the most important question is that most of the above studies have a premise that the subjects need to undergo a lot of training. Undeniably, if animals can acquire the ability of BPS after training, it can solve some evolutionary puzzles, such as if animals can learn BPS, then music does not need to consider BPS in natural selection. But does this species have the ability of BPS innate? During the training process, the researcher’s goal is to teach the animal the rules of the experiment, but there is also an opportunity to allow the animal to learn other abilities that do not exist in genetic evolution, which is difficult to distinguish. In a recent study, it was found that rats can synchronize their movements to the beat of music just like humans, without any motive to move (Ito et al., 2022). Rodents are not mammals that belong to vocal learning, and there is no species closely related to them that can perform vocal learning. This finding poses a significant challenge to the vocal learning hypothesis. As described by the vocal learning hypothesis, only species that can perform vocal learning can produce rhythm synchronization. However, rodents that lack vocal learning abilities can also spontaneously produce rhythm synchronization movements. Does this imply that rodents might be an undiscovered type of vocal learning species, or does it suggest that the vocal learning hypothesis — which posits that auditory input can regulate general motor behavior (not just vocal motor behavior) — is overly simplistic when taken literally?

The most important factor for the generation of beat perception and synchronization is the need for a species to have the ability to time and predict events. Studies on humans have shown that the cortex-cerebellum can measure intervals within the range of 100–2000 ms accurately, while supra-second range time is believed to be based on interactions between cortical areas, basal ganglia, and thalamus (Petter et al., 2016). Brain regions involved in time perception and timing performance have also been identified through human neuroimaging studies (Lewis and Miall, 2003; Meck et al., 2008; Coull et al., 2011; Merchant et al., 2013a; Allman et al., 2014). In duration discrimination or reproduction tasks, significant activation has been commonly reported in the cortical-basal ganglia-thalamus loop (including the DLPFC, supplementary motor area (SMA), preSMA, striatum, and thalamus), the ventral striatum, and the cerebellum (Lewis and Miall, 2003; Rubia and Smith, 2004; Meck et al., 2008). Lesion and inactivation studies have found that damage to the caudate/putamen (CPu) and the loss of dopamine (DA) in the substantia nigra pars compacta (SNc) typically result in significant deficits in temporal control in humans and rodents (Meck, 2006b; Jones and Jahanshahi, 2011; Schwartze et al., 2011; Schwartze and Kotz, 2013; Cope et al., 2014; Yin et al., 2022). Clinical populations exhibit deficits in the cortical-basal ganglia-thalamus loop and DA regulation of timing; evidence of timing abnormalities has been observed in attention deficit hyperactivity disorder (ADHD), Huntington’s disease (HD), Parkinson’s disease (PD), schizophrenia, autism, and obsessive-compulsive disorder (OCD) (Beste et al., 2007; Carroll et al., 2008; Jahanshahi et al., 2010; Gu et al., 2011; Cope et al., 2014). Damage to the frontal cortex (FC) can also affect interval timing, for example, by reducing the influence of dopamine agonists on clock speed (Meck, 2006a). Specifically, patients with lesions to the right FC and ventral prefrontal cortex exhibit impaired time processing (Harrington et al., 1998), suggesting a critical role for the right hemisphere in timing. Repetitive transcranial magnetic stimulation (TMS) of the right dorsolateral prefrontal cortex (DLPFC) also produces time impairments lasting several seconds (Koch et al., 2003; Jones et al., 2004; Wiener, 2014), further supporting the involvement of the right hemisphere in supra-second timing. In rodents, damage or inactivation of the medial prefrontal cortex (mPFC) typically results in many premature responses, terminating ongoing responses before the end of the delay period in lever-pressing experiments (Narayanan et al., 2006). The explanation for this phenomenon is that the medial prefrontal cortex (PFC) encodes information related to the passage of time and sends this information to the motor cortex, where inappropriate actions are inhibited (Narayanan and Laubach, 2006; Merchant et al., 2013b).

The auditory system also plays a crucial role in BPS, with rhythm perception being associated with functional coupling between auditory and motor regions. Grahn and Rowe (2009) investigated rhythm perception in musicians and non-musicians using functional magnetic resonance imaging (fMRI), discovering that beat presence correlated with increased connectivity between the putamen, supplementary motor area (SMA), premotor cortex (PMC), and auditory cortex. Patel and Iversen (2014) proposed the Action Simulation for Auditory Prediction (ASAP) hypothesis, suggesting that beat perception necessitates temporally precise bidirectional communication between auditory regions and motor planning regions. This hypothesis posits the existence of a “posterior auditory pathway” that connects the posterior superior temporal gyrus (pSTG) with the dorsal premotor cortex (dPMC) through the parietal lobe, facilitating time-accurate signal transmission between auditory and motor planning regions. During rhythm perception, functional connections between motor and auditory regions enable motor planning signals to influence auditory processing and perception. However, the ASAP hypothesis also implies that this “posterior auditory pathway” might differ between humans and other primates, potentially due to the evolutionary impact of vocal learning in the human lineage. A neural anatomy study reported sparse projections of the STG and pSTG in macaques (Lewis and Van Essen, 2000), suggesting that projection intensity in this region could represent a significant neural anatomical distinction between humans and macaques. Merchant and Honing (2014) posited that differences in neural pathways between the temporal cortex and premotor cortex across species might explain the inferior synchronization with periodic stimuli observed in monkeys compared to humans. Further research is needed to determine whether these neural differences are indeed responsible for variations in beat perception among humans and other primates. Moreover, long-term studies have indicated that individuals generally exhibit superior performance in beat perception with auditory stimuli compared to visual stimuli (Iversen and Balasubramaniam, 2016). Sensory-motor synchronization (SMS) to temporally discrete auditory and visual stimuli has consistently demonstrated an auditory advantage, attributable to differential connectivity among auditory, visual, and motor systems (Patel et al., 2005; McAuley and Henry, 2010). Nonetheless, some research has shown that periodically moving “bouncing” visual stimuli can drive discrete (tapping) synchronization with accuracy similar to or equaling auditory beeps (Hove et al., 2013; Iversen et al., 2015). A study involving congenitally deaf individuals revealed that the long-term observed auditory advantage in rhythm synchronization heavily relies on stimulation and experience (Iversen et al., 2015). In some cases, silent moving visual stimuli can drive synchronization as accurately as sound, and a purely visual stimulus can elicit discrete rhythmic synchronization nearly as accurately as an auditory metronome in the general population. However, a macaque study found that while these primates can synchronize visually, they display reduced sensitivity in the auditory domain (Merchant and Honing, 2014). Consequently, when examining non-human species’ ability to perceive and synchronize to rhythms, it is essential to consider various stimuli types that may impact this capacity, as well as the coupling between different sensory and motor regions in the brain that could contribute to BPS.

As previously discussed, neuroscience studies on rodents, in addition to primate research, reveal their capacity for interval timing, with human brain imaging experiments confirming the cortical-basal ganglia-thalamic circuit’s primary role in this ability. This evidence calls for further scrutiny of the vocal learning hypothesis’s validity, suggesting that the hypothesis may need to be revised, while acknowledging the importance of the interaction between auditory and motor systems in BPS. Concurrently, the methodological limitations of visual inspection, upon which the hypothesis and many BPS studies are based, should be recognized, as previously illustrated. Furthermore, the influence of other relevant brain regions on interval timing warrants increased attention to these areas during cross-species investigations of rhythm perception synchronization. This approach can help determine whether BPS ability is a common trait among species, stems from ancient common ancestors, or presents evolutionary differences unique to a select few species.

**FIGURE 1:**
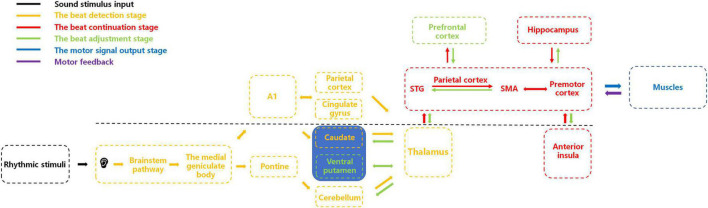
An integrative brain-circuit model of beat perception and synchronization. This figure illustrates the activation of corresponding brain regions and their interactions during different stages of beat perception and synchronization. The primary auditory cortex (A1) is the primary sensory area for auditory perception, while the superior temporal gyrus (STG) and the supplementary motor area (SMA) play important roles in auditory-motor integration. In the beat detection stage, parietal cortex and cingulate gyrus are activated, focusing individual attention on auditory stimuli. The putamen and caudate receive auditory signals from MGB and pons, respectively, for beat detection. In the beat continuation stage, auditory signals provided by other brain regions are passed to the basolateral auditory pathway (STG, parietal cortex, and SMA, premotor cortex) through the thalamus for beat continuation. The hippocampus retrieves previous memories to provide relevant experience for beat continuation. The anterior insula integrates relevant information. In the beat adjustment stage, when the beat changes, the ventral putamen receives relevant beat information from the thalamus and recalculates it, passing reorganized information to the basolateral auditory pathway. The prefrontal cortex performs cognitive evaluation, and the anterior insula integrates relevant information again. The hippocampus stores these experiences in memory. Muscles receive motion signals from the brain, perform related movements, and provide feedback on the results to the brain. Inputs to the striatum and cerebellum mainly come from the cortex through the thalamus, which serves as a hub for relaying information between different brain regions.

Here, as illustrated in Figure 1, we present a potential integrative neural-circuit model of beat perception and synchronization informed by previous neural and physiological studies (Budinger and Scheich, 2009; Rauschecker, 2011; Patel and Iversen, 2014; Hackett, 2015; Kumar et al., 2016; Rajendran et al., 2017; Zuk et al., 2018; Cameron and Grahn, 2021; Cannon and Patel, 2021; Kasdan et al., 2022). This model comprises five stages: sensory input, beat detection, beat continuation, beat adjustment, and motor synchronization. We propose that after processing auditory sensory stimulation through the primary auditory system, the beat detection stage commences, involving neural interactions among subcortical and cortical brain regions to detect the beat. Upon beat detection, information is relayed via the thalamus to the basolateral auditory pathway (encompassing the superior temporal gyrus, parietal cortex, and supplementary motor area) for beat continuation, with simultaneous involvement of brain regions such as the premotor cortex, hippocampus, and anterior insula. As external auditory stimuli change, areas like the ventral putamen and prefrontal cortex become activated, adjusting their corresponding regions. Subsequently, structures responsible for motor control, such as the cerebellum, regulate limb movements for synchrony. While substantial evidence and hypotheses have been explored in humans regarding BPS, comparative studies can be conducted in other species to determine if similar BPS characteristics exist across species. We hope our proposed model offers valuable guidance for cross-species research, enabling exploration of BPS presence and potential differences among various species. Investigating these questions will further our understanding of the physiological mechanisms of BPS and the neural and physiological connections across species. Simultaneously, this model may serve as a valuable inspiration for rhythm perception research, promoting advancements in this field.

## Conclusion

Currently, some researchers believe that musicality is not a single trait that evolved to solve a specific problem (such as infant emotional regulation or sexual attraction), but rather a set of abilities that can be used in different ways to support multi-functionality, all of which involve social belonging (Savage et al., 2021). In other words, the purpose of music is to facilitate social bonding and establish and reinforce personal affiliations. The Musicality and Social Bonding (MSB) hypothesis posits that the core biological component of human musicality evolved as a mechanism to support social bonding, and social bonding is the ultimate functional explanation for the evolution of musicality (Savage et al., 2021). Most music has two unique rhythmic components: isochronous (evenly timed) beats and rhythmic structure (Arom, 1991; London, 2012; Savage et al., 2015). These core design features of musicality do not appear to be designed for solo performance, but rather support synchronized and coordinated musical sounds and group dance movements, which are universal features of the human musical system (Savage et al., 2015). The MSB hypothesis also proposes a putative neural biology approximation mechanism that supports the social impact of music. Relevant brain areas, such as the basal ganglia (BG), ST (superior temporal lobe structures), Motor (frontal lobe structures), and vmPFC (ventromedial prefrontal cortex), are all strikingly consistent with the brain regions found in studies of beat perception and synchronization. On the other hand, some researchers argue that it is necessary to compare temporal perception in social and non-social environments and to explore social perception when manipulating time-related factors such as rhythm and speed (Schirmer et al., 2016). Such research will not only deepen our understanding of the social meaning of time, but also provide insights into the more general relevance of beat-perception-based musicality in human sociality.

Therefore, in future cross-species studies of musicality, the social bonding attributes of musicality can be considered, and more exploration can be conducted to discover whether gregarious vertebrates have similar abilities related to musicality, as revealed by rodents. In addition to studying musicality, the question of whether other species enjoy music is also worth exploring. A study found that chimpanzees have preferences for different types of music, and this response is not a result of novelty (Mingle et al., 2014). Based on this finding, further research can investigate why chimpanzees have these preferences, what kind of experience specific types of music can provide for organisms, and whether they can have specific effects such as soothing emotions and calming, similar to humans.

In summary, cross-species research on music can help us elucidate the evolutionary history of musical cognition, understand how other species have evolved and developed on the path of evolution, and gain a better understanding of the development and changes of humans in terms of music. This field requires further development and research from different types of researchers.

## Author contributions

BY initiated and supervised the project and provided critical revisions. J-KH researched and wrote the initial manuscript. Both authors agreed to the final version of the manuscript.
